# HIV Treatment as Prevention: Optimising the Impact of Expanded HIV Treatment Programmes

**DOI:** 10.1371/journal.pmed.1001258

**Published:** 2012-07-10

**Authors:** Wim Delva, Jeffrey W. Eaton, Fei Meng, Christophe Fraser, Richard G. White, Peter Vickerman, Marie-Claude Boily, Timothy B. Hallett

**Affiliations:** 1 South African Department of Science and Technology/National Research Foundation Centre for Excellence in Epidemiological Modelling and Analysis, University of Stellenbosch, Stellenbosch, South Africa; 2 International Centre for Reproductive Health, Ghent University, Ghent, Belgium; 3 Department of Infectious Disease Epidemiology, Imperial College London, London, United Kingdom; 4 Center for Statistics, Hasselt University, Diepenbeek, Belgium; 5 Department of Infectious Disease Epidemiology, London School of Hygiene & Tropical Medicine, London, United Kingdom; 6 Department of Global Health and Development, London School of Hygiene & Tropical Medicine, London, United Kingdom; Duke University Medical Center, United States of America

## Abstract

Until now, decisions about how to allocate ART have largely been based on maximising the therapeutic benefit of ART for patients. Since the results of the HPTN 052 study showed efficacy of antiretroviral therapy (ART) in preventing HIV transmission, there has been increased interest in the benefits of ART not only as treatment, but also in prevention. Resources for expanding ART in the short term may be limited, so the question is how to generate the most prevention benefit from realistic potential increases in the availability of ART. Although not a formal systematic review, here we review different ways in which access to ART could be expanded by prioritising access to particular groups based on clinical or behavioural factors. For each group we consider (i) the clinical and epidemiological benefits, (ii) the potential feasibility, acceptability, and equity, and (iii) the affordability and cost-effectiveness of prioritising ART access for that group. In re-evaluating the allocation of ART in light of the new data about ART preventing transmission, the goal should be to create policies that maximise epidemiological and clinical benefit while still being feasible, affordable, acceptable, and equitable.

## Introduction

There has been a rapid expansion in access to antiretroviral therapy (ART) over the past decade, especially in the countries with the highest burden of HIV. At the end of 2010, an estimated 6.7 million people were on ART globally, an increase of over 1.4 million from the previous year, but around 7.5 million people are still in need of treatment based on current World Health Organization (WHO) guidelines [1]. Until now, decisions around how to allocate ART have been based on maximising the therapeutic benefit of ART for patients, within the constraints of limited financial and health care system resources [2]. This has led to ART access being prioritised for those with the lowest CD4 cell counts (and patients with active tuberculosis [TB]) [3].

The HPTN 052 study [4] demonstrated that earlier ART initiation can reduce heterosexual HIV transmission [5]. This finding suggests that future expansions of ART access should seek to maximise not only the therapeutic but also the prevention benefits of treatment. Currently, constrained resources and capacity for HIV treatment and prevention [6]–[8] make it unfeasible to immediately provide ART for all people living with HIV, even if this was the optimal epidemiological and therapeutic strategy and was widely accepted by communities. However, as increasingly high levels of access under current guidelines are achieved in coming years, the recent information about the prevention benefit of ART has inspired renewed discussion about whether and how to incrementally expand access to treatment to subgroups that will differentially benefit from the preventive and therapeutic features of ART, especially in sub-Saharan Africa, where the burden of HIV is greatest.

Candidate priority groups for early treatment are defined by both clinical and behavioural criteria. Potential clinical criteria for providing early treatment include the following: incrementally increasing the CD4 cell count threshold for treatment eligibility, immediate treatment for those with high set-point viral load, immediate treatment for pregnant women, and immediate treatment for those with TB coinfection. Behavioural risk groups that have been proposed for early treatment include HIV-serodiscordant couples, female sex workers (FSWs), men who have sex with men (MSM), and people who inject drugs (PWID). Expanding access to treatment for each of these subgroups is evaluated here according to (i) clinical and epidemiological benefits, (ii) potential feasibility, acceptability, and equity, and (iii) affordability and cost-effectiveness (Box 1).

**Box 1. A Breakdown of Questions Related to the Impact, Feasibility, Affordability, and Acceptability of Expanded ART Provision**

**Epidemiological Impact**

- What is the incremental effectiveness of the expanded ART programme for averting new HIV infections, relative to the existing HIV prevention and treatment programme?
- What is the likelihood of behavioural risk substitution that could undermine prevention benefits?

**Clinical Impact**

- What is the incremental effectiveness of expanding ART for averting HIV-related morbidity and mortality, relative to the existing modes of ART delivery?
- What is the potential impact of expanded access to ART on the acquisition and transmission of drug resistance?

**Affordability and Cost-Effectiveness**

- What size is the additional priority group?
- What is the expected start-up cost of the expanded ART programme?
- How would the programme costs accumulate over time?
- Would the programme be cost-effective compared to accepted international benchmarks, and relative to alternative HIV prevention methods?

**Feasibility**

- What infrastructure and human resources does the expanded ART programme require?
- How would the prioritisation for a particular group be operationalized?
- What is the expected adherence and retention in care for the additional priority group?

**Acceptability**

- Would the expanded ART programme violate principles of health ethics or human rights?
- Would the expanded ART programme be acceptable to the newly eligible priority group, communities, and decision-makers?

This article is not a systematic literature review of all clinical, epidemiological, and policy implications of alternative options for expanding HIV treatment programmes. Rather, it represents an organised collection of expert opinions, literature reviews, and multidisciplinary discussions. Following the publication of the HPTN 052 results and the US President's Emergency Plan for AIDS Relief (PEPFAR) Scientific Advisory Board recommendations for PEPFAR HIV treatment programmes [2], experts in the field of HIV epidemiology, mathematical modelling, and HIV policy were convened in an HIV Modelling Consortium (http://www.hivmodelling.org) meeting in November 2011 to discuss the potential impact of expanded HIV treatment in sub-Saharan Africa. Following from this meeting, this review focuses on the biological and behavioural factors that determine the potential impact of various ART prioritisation options on transmission, morbidity, and mortality, as well as the factors affecting feasibility, affordability, acceptability, cost-effectiveness, and health systems interactions. These include the relative size of the priority group, anticipated ease of identification and recruitment of the priority group, treatment uptake, adherence and loss to follow-up, ethical challenges, and technical and human resources required.

## Potential Prioritisation Groups for ART Expansion CD4 Cell Count

### CD4 Cell Count

**Figure 1:**
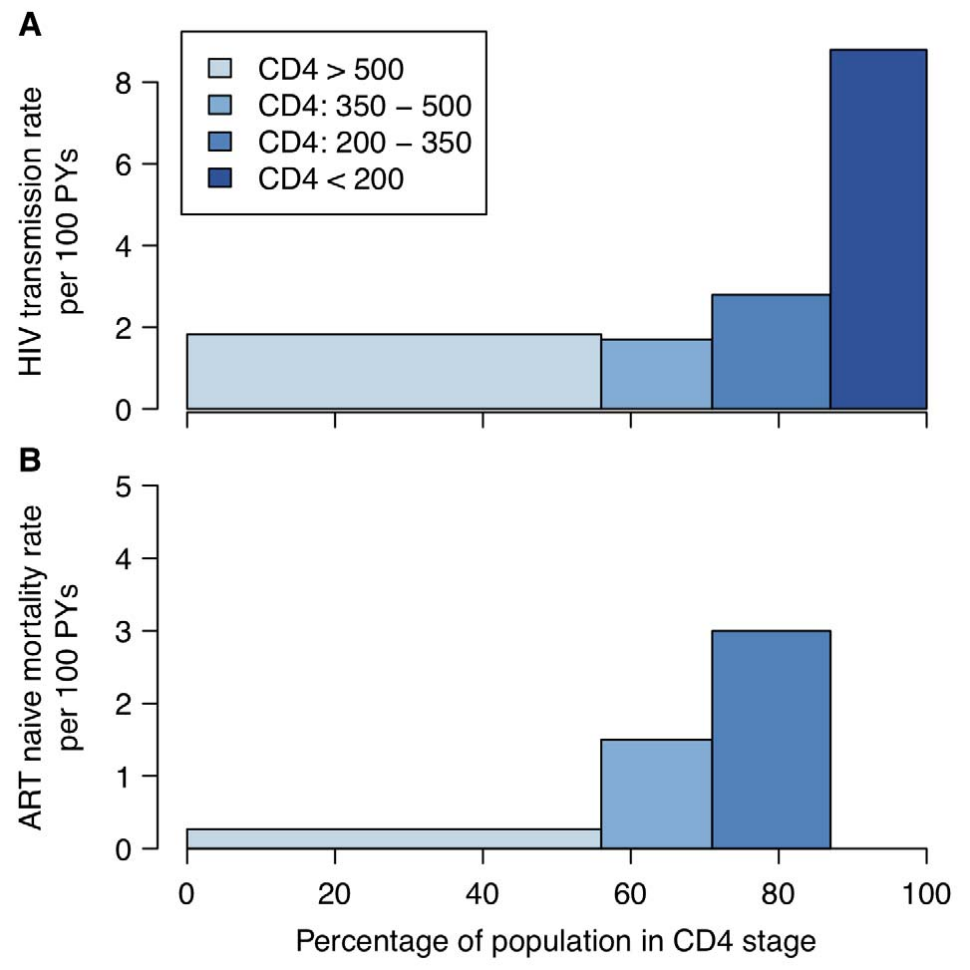
HIV transmission and mortality by CD4 count. (A) HIV transmission rate per 100 person-years (PYs) by CD4 count for the infected partner in discordant couples enrolled in a randomized controlled trial of acyclovir [11]. (B) Mortality rate by CD4 category in ART-naïve HIV-positive individuals enrolled in research cohorts in West Africa [132]. In both panels, the width of the bars represents the proportion of ART-naïve HIV-positive 15- to 64-year-olds by CD4 count in a nationally representative household survey in Kenya [15].

As many low- and middle-income countries are moving towards adoption of the WHO guidelines of providing treatment for all HIV-infected individuals with CD4 cell counts less than 350 cells/µl [3], one natural strategy for increasing the prevention benefit of treatment is to further increase the threshold of eligibility to those with CD4 counts less than 500 cells/µl. Current US treatment guidelines recommend initiation of treatment for asymptomatic HIV-infected individuals with CD4 counts between 350 and 500 cells/µl [9], and European guidelines suggest that treatment should be considered at this point [10]. Observational and clinical trial data that link transmission events confirm that heterosexual transmissions occur from asymptomatically infected individuals with CD4 counts between 350 and 500 cells/µl [5],[11], and the HPTN 052 study demonstrated a 96% reduction in transmission associated with treatment initiation at a CD4 cell count between 350 and 550 cells/µl compared to delaying treatment until CD4 count was below 250 cells/µl [5]. However, compared to other CD4 strata, individuals with CD4 counts between 350 and 500 cells/µl have the lowest transmission rates [11] (Figure 1), suggesting that expanding treatment to this group without considering other biological or behavioural transmission risk factors may be the least efficient strategy for prioritising treatment for prevention.

The magnitude of the overall long-term additional therapeutic benefit of providing treatment at CD4 count above 350 cells/µl is uncertain. A collaborative analysis of observational data found that deferring treatment initiation from between 351 and 450 cells/µl to between 251 and 350 cells/µl increased the hazard of AIDS or death by 28% [12], and the HPTN 052 trial found that delaying treatment until CD4 count was lower than 250 cells/µl was associated with a 41% increased hazard of adverse clinical outcome [5]. However, the potential benefits of early treatment need to be weighed against the potential toxicities of ART and negative effects on quality of life [9]. Earlier treatment initiation may also be associated with poorer adherence or retention in care [13], which can lead to increased risk of drug-resistant virus. More robust data about the clinical benefit of earlier treatment and patients' retention in care when treatment is initiated earlier are expected from the START trial [14].

Both the cost and epidemiological impact of expanding eligibility for ART to those with CD4 counts up to 500 cells/µl will largely be determined by the number of additional people on treatment. Cross-sectional data from sub-Saharan Africa suggest that between 20% and 25% of HIV-infected people have CD4 counts between 350 and 500 cells/µl [15]. Based on the Joint United Nations Programme on HIV/AIDS estimate of approximately 19.8 million adults infected in sub-Saharan Africa [16], increasing the CD4 threshold would add between 4 and 5 million to the 10 million people currently still in need of treatment.

However, even with a change in the threshold at which patients are considered eligible for treatment, the numbers expected to initiate treatment at high CD4 counts will be low without improvements in frequency of testing and retention in pre-ART care [17]. Surveillance of HIV testing programmes in a township near Cape Town, South Africa, found that amongst individuals accessing voluntary counselling and testing, 66% of those testing HIV-positive already had CD4 cell counts below 350 cells/µl [18]. Another testing-related problem is that within-patient variability in CD4 cell count can be very high [19], such that the CD4 count from a single test could be an unreliable indicator of transmission risk and clinical need [20],[21]. Moreover, HIV-infected individuals who are feeling healthy may decline the option to initiate treatment [22], a challenge likely to be exacerbated under earlier treatment eligibility at high CD4 counts. Earlier access to ART would, on the other hand, also reduce the number of patients needing pre-ART care, the phase at which retention is the poorest, according to a systematic review of retention in HIV care in sub-Saharan Africa [17].

Steadily increasing the CD4 threshold for treatment eligibility as further resources become available may be viewed as the most equitable and acceptable strategy for allocating additional treatment, considering that treatment eligibility has long been based on a CD4 criterion, but while resources for treatment continue to be constrained, expanding treatment access beyond current clinical guidelines based on an increasing CD4 criterion is unlikely to be the most efficient route to maximising the epidemiological or clinical benefit of ART.

### Viral Load

Untreated asymptomatic HIV infection is characterised by the viral load fluctuating around a steady level, called the set-point viral load (SPVL) [23]. Individuals vary considerably in SPVL; values are approximately log-normally distributed with standard deviation 0.75 log_10_ units, such that the 95% range spans a 1,000-fold variation in SPVL [24]. SPVL has proven one of the more robust predictors of infectiousness [11],[25]–[28]. In a recent study amongst serodiscordant couples [28], the transmission rate in couples with index individuals with viral load in the range 100,000 to 1,000,000 copies/ml of blood was estimated to be 5.6 per 100 person-years at risk (95% confidence interval: 4.0 to 7.6), while the transmission rate for index individuals with viral load in the range 100 to 1,000 copies/ml was estimated at 0.8 per 100 person-years (0.4 to 1.5). Thus, a 100-fold difference in viral load translates to a 7-fold variation in infectiousness, although this relationship is highly nonlinear [11],[25]–[28]. As it becomes easier to measure viral loads in the field, with point-of-care tests in development (e.g., [29]), it becomes reasonable to ask whether prioritising further ART expansion for individuals with high viral load would be an effective, efficient, and affordable strategy.

**Figure 2:**
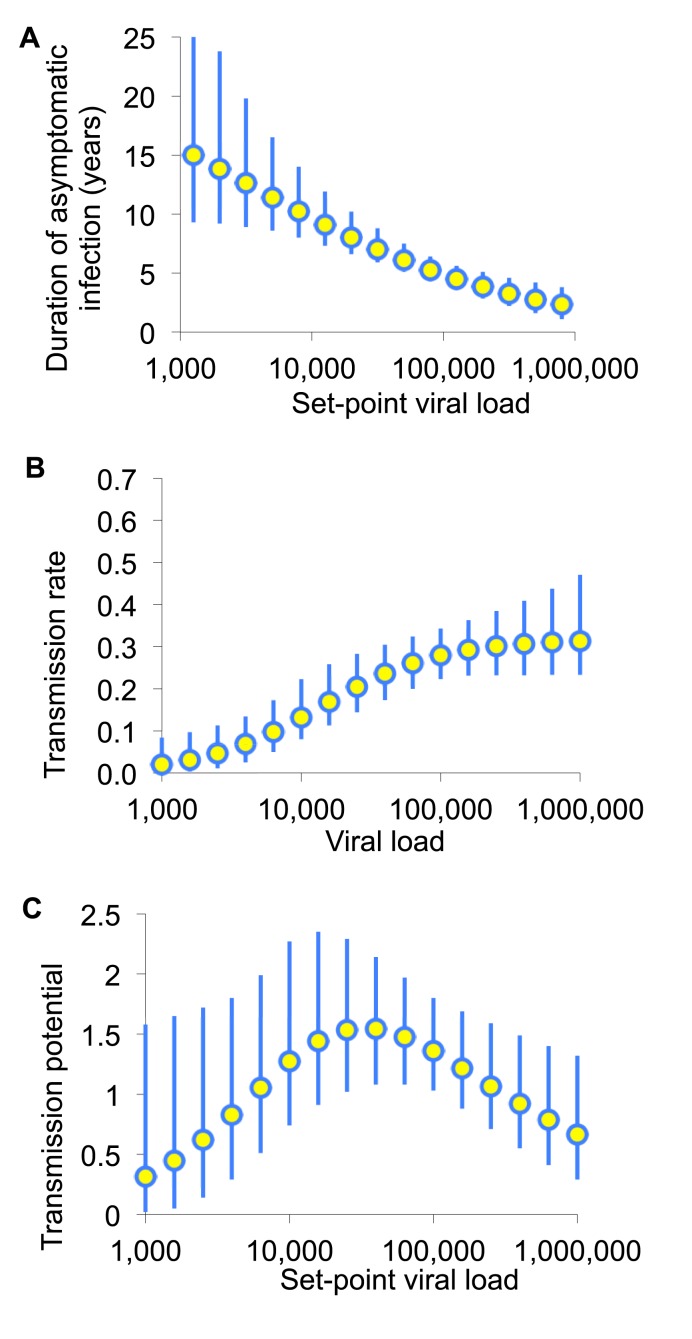
The transmission potential of individuals as a function of set-point viral load. (A) Infectiousness (per unit calendar time) and (B) duration of asymptomatic infection are estimated by fitting to various sources of data as described in [24]. (C) The product of these is the transmission potential, the average number of people an infected individual is expected to infect over the whole of asymptomatic infection. The transmission potential measures the relative prevention effect of treatment as prevention targeted to an individual with a given SPVL. Adapted from Fraser et al. [24].

While individuals with high SPVL are more likely to effectively transmit the virus, they also tend to progress from asymptomatic infection to disease more quickly than those with low SPVL [30]. To estimate how much individuals with differing SPVL contribute to the epidemic, their transmission potential can be calculated as the product of their biological infectiousness and duration of infection [24] (Figure 2). Compared to individuals with intermediate SPVL, individuals with very high SPVL may contribute less to the epidemic, because they progress to advanced disease and death very quickly and thus have fewer opportunities to infect others.

Consequently, prioritised ART expansion for individuals with very high viral loads may not provide greater long-term prevention benefits than expanded access for a comparably large random fraction of the untreated population. The principal frailty in this conclusion comes from multiplying infectiousness and duration of asymptomatic infection from different studies. However, this conclusion is robust to parametric assumptions, to assumptions about the sexual network, and to including heightened infectiousness in early- and late-stage untreated infection [24],[31].

While the epidemiological benefit of expanded ART access for individuals with very high SPVL may be limited, targeting these individuals for rapid ART initiation may offer substantial clinical benefits. ART prioritisation for people with very high SPVL could be expensive to implement, as viral load screening and follow-up would require substantial resources. How this form of prioritisation would affect the number of patients eligible for treatment is not clear: a recent analysis of HIV-1 RNA viral load data from two general population cohorts in Botswana suggested that 24%–28% and 14%–18% of HIV-infected, treatment-naïve individuals (*n* = 1,286) had viral load levels greater than 50,000 and 100,000 copies/ml, respectively [32], but it is unclear how many of these individuals were not eligible under current CD4-based ART initiation guidelines.

### Pregnant Women

Existing guidelines for the prevention of mother-to-child transmission (PMTCT) recommend that pregnant women with CD4 counts higher than 350 cells/µl take an antiretroviral drug course from the 14th week of pregnancy until one week after delivery (Option A) or until one week after breastfeeding has finished (Option B) [33]. A new option “B+” has been proposed, in which pregnant women would be eligible to immediately initiate lifelong ART regardless of HIV disease stage, TB disease status, or CD4 count [34]. The cost and epidemiological impact of expanding ART to all pregnant women will vary between settings with different patterns of fertility, sexual behaviours, and existing ART programmes.

The potential HIV prevention impact of option B+, beyond PMTCT, would be low if many infected pregnant woman are in stable relationships with partners who are already infected. Data from Demographic and Health Surveys in Lesotho, Malawi, and Kenya indicate that more than half of married, cohabiting partners of HIV-infected pregnant women are HIV-infected (83% [10/12] in Lesotho, 54% [20/37] in Malawi, and 50% [6/12] in Kenya) [35]–[37]. However, for serodiscordant couples, the female-to-male transmission rate may be more than twice as high during pregnancy as during non-pregnant periods [38]. Whether ART initiation during pregnancy would effectively override this risk elevation is questionable, given the lag time of up to five months between ART initiation and viral load suppression [39].

The number of additional people who would be on treatment with this prioritisation strategy depends on several factors. The crude birth rate (and hence the incidence of pregnancy) varies greatly across sub-Saharan Africa, even within subregions: from as high as 46.5 childbirths per 1,000 people per year in Zambia to less than half this rate (22.9/1,000 individuals/year) in the neighbouring country of Botswana [40]. However, overall, the difference between the number of HIV-infected pregnant women that would be ART-eligible under PMTCT option B+ and the number eligible under current ART initiation guidelines may be small because of the effect of haemodilution on CD4 cell count. Haemodilution, a normal physiological phenomenon during pregnancy, temporarily reduces CD4 cell count, meaning that many pregnant HIV-infected women become eligible for treatment on the basis of CD4 count during pregnancy. In a prospective cohort study of 324 HIV-infected pregnant women from Abidjan, Cote d'Ivoire, 48.3% (157/325) had CD4 counts less than 350 CD4 cells/µl at 32 weeks of gestation, yet this fraction decreased to 28.9% (94/325) one month postpartum [41]. The implication for Cote d'Ivoire is that only just over 10,000 additional women would initiate ART if immediate treatment was expanded to all pregnant women regardless of CD4 count, and ART coverage in women, in the first year of the intervention, would increase only from 39% to 42% [42].

HIV-positive pregnant women are a priority group that is relatively easy to identify, because of the high uptake of antenatal care (ANC) in most populations, with associated HIV counselling and testing, even in resource-limited settings. Several studies reported very high acceptance of provider-initiated HIV counselling and testing in ANC at several sites across Africa during the past few years: 99.5% acceptance of testing in Nigeria, 91% in South Africa, 97% in Ghana, and 99% in Zambia [43]–[46]. The acceptance of HIV testing at first ANC visit is still as low as 69.1% in rural areas of Swaziland and South Africa [47], but provider-initiated testing and counselling in ANC may be able to raise the testing uptake by 9.9%–65.6% [48].

Obstacles remain in the linkage between diagnosis in ANC and long-term ART treatment because of ART refusal and poor retention. In a recent review, Ferguson et al. found that 38%–88% of known ART-eligible women in sub-Saharan countries fail to initiate treatment [49]. Once in treatment, retention among pregnant women has been found to be no worse than in other population groups in seven resource-limited countries in sub-Saharan Africa and Thailand [50]. However, Boyles et al. found that initiating ART while pregnant is associated with a higher lost-to-follow-up risk compared with the general population in rural South Africa [51]. Retention challenges faced when expanding ART to pregnant women regardless of CD4 count are likely to be similar to those currently faced in traditional PMTCT programmes: (1) patients' not being prepared for HIV testing and its implications before the ANC visit; (2) fear of stigma, discrimination, household conflict, or divorce on disclosure of HIV status; (3) long waiting times at the ANC facilities; and (4) inability to afford the transport to these facilities [52].

Because expanding access to ART for pregnant women utilises existing ANC and PMTCT infrastructure for diagnosis and HIV counselling, the only additional costs associated with this strategy are additional drug costs for the period between the end of pregnancy and ART eligibility under other criteria, suggesting favourable affordability of this ART expansion strategy. Cost-effectiveness studies of ART in pregnant women have thus far focused on benefits in terms of PMTCT, and have found that it is cost-effective as measured against accepted international benchmarks in a variety of low- and middle-income countries [53],[54]. Cost-effectiveness studies of PMTCT option B+ for adult HIV transmission prevention are still to be conducted. Expanding ART to all HIV-positive pregnant women may provide additional maternal health benefits and contribute to the Millennium Development Goals if ART, PMTCT, and reproductive health care services are integrated [55],[56].

### Active Tuberculosis Disease

The provision of ART to all HIV-infected people with active TB disease, irrespective of CD4 cell count, has been recommended by the World Health Organization since 2010 [3], based on its clinical benefits. In the SAPiT trial, the mortality rate in 429 patients with CD4 cell counts up to 500 cells/µl who initiated ART during TB treatment was 56% lower (95% confidence interval: 21%–75%, *p* = 0.003) than in patients who initiated ART after completion of TB treatment [57]. However, the coverage of ART for all HIV-infected people with active TB disease remains low. In the WHO African Region in 2011, only 59% of TB patients were tested for HIV, and of those identified to be HIV-infected, only 42% were on, or started on, ART [58].

The epidemiological benefits of expanding ART to all patients with active TB disease are unclear. Although there were some early indications that those with TB disease are more infectious [59]–[61], the largest study conducted among HIV-positive people with incident TB disease indicated that viral load increases by only a small amount following a TB episode [62], and a more recent study showed that treating active TB disease in individuals with CD4 counts greater than 350 cells/µl reduced markers of immune activation but had no effect on HIV viral load or CD4 count [63]. Therefore, providing ART to TB patients with CD4 counts above 350 cells/µl is likely to have a similar prevention effect on HIV transmission as treating a random subset of HIV-infected individuals with a CD4 count above 350 cells/µl.

In high HIV prevalence settings, the proportion of HIV-infected people with active TB who have CD4 cell counts greater than 350 cells/µl has been estimated to range from 11% to 30% [2]. Assuming a 1% incidence of active TB disease and 50% HIV prevalence in individuals with incident active TB, this would mean that for South Africa, around 27,500 to 75,000 extra individuals would be eligible for ART in the first year of this form of prioritisation. Given the suppressive effect of ART on TB disease incidence [64],[65], a decreasing number of active TB patients in need of ART would be expected in the following years. A modelling study that estimated the impact of the roll-out of annual HIV testing and immediate ART on TB disease incidence in nine African countries reported a 21% (range: 10%–31%) reduction in the cumulative AIDS-related TB disease incidence over the first five years, and a 48% (range: 37%–55%) reduction in the incidence of TB disease at five years [64].

Integration of ART provision for all HIV patients, regardless of TB coinfection status, with TB services may offer a relatively feasible way to implement an expansion of ART to individuals with active TB disease. Data from eight countries with a high burden of HIV-infection-associated TB showed that there were up to five TB treatment facilities for each ART facility in 2007 [66], and a study in Tugela Ferry, South Africa, showed that integration of TB and HIV services was associated with high ART adherence [67]. However, with this approach it would be critical to implement adequate infection control to minimise nosocomial TB infection, and obtaining high TB treatment coverage is challenged by the difficulty of diagnosing active TB in HIV-infected patients [68].

Given the clear clinical benefit of ART in TB patients, this option of ART expansion is likely to be highly acceptable by both the target group and the general population. For TB patients, current illness and the prospect of a reduced risk of TB recurrence are incentives for ART initiation, high adherence, and retention in care. If implemented successfully, ART expansion to all TB patients should lead to large gains in healthy person-years of life and long-term cost savings due to decreased recurrent TB.

### Serodiscordant Long-Term Relationships

Stable serodiscordant relationships, in which one partner is HIV-infected and the other is not, are an identifiable prevention opportunity, and the continued transmission in such couples during carefully monitored clinical trials with intensive counselling demonstrates the need for additional prevention options for this population [5],[11]. Trial and observational data have demonstrated the efficacy of ART in preventing HIV transmission in stable serodiscordant heterosexual partnerships [5],[11], and recent WHO guidelines for stable serodiscordant couples already include offering ART to the HIV-infected partner irrespective of CD4 cell count, in addition to behaviour change counselling [69]. While the biological efficacy of the effect of ART on transmission risk should generalise to non-stable heterosexual partnerships as well, it has been hypothesized that couples in stable partnerships will be most able to adhere to daily dosing regimens and therefore achieve the maximum individual-level benefit [70],[71]. Further, it is known that couples in stable partnerships in which the HIV-infected individual has a high CD4 cell count are likely to conceive (16% per year among discordant couples [38]); therefore this strategy would incur many of the maternal and PMTCT-related health benefits described above [33].

The relative epidemiological impact of prioritising early treatment to HIV-infected individuals who have an uninfected long-term partner will depend primarily on the risk of within-couple transmission without treatment, and secondarily, on the risk of onward transmission from the partner to someone else. The risk of transmission without ART in couples could be relatively low: 1.7 per 100 person-years at risk ([5]; among those with CD4 counts of 350–500 cells/µl: [11]). One model suggests that providing ART to serodiscordant couples might be expected to avert 21 infections per 1,000 person-years of ART [72]. If the risk of transmission in couples is actually higher (as has been observed in couples that did not necessarily know that they were in a discordant partnership [73], and as assumed by El-Sadr et al. [74]), and if it is assumed that the infected partner forms many additional partnerships with other individuals, then it has been estimated that the number of infections averted per 1,000 person-years of ART could be as high as 77. This can be compared to 53–159 for providing ART to all individuals with CD4 cell counts below 350 cells/µl irrespective of partnership status, and 65–152 if ART is provided irrespective of CD4 cell count [75]. Thus, prioritising those in stable partnerships for treatment may not be a more efficient form of prevention than providing the treatment to the general population without prioritisation.

It is unclear how feasible it would be to preferentially provide access to ART to those in serodiscordant couples. Only ∼8%–31% of couples were found to be discordant in recruitment to a clinical trial [76], and other data suggest that the countries with the highest levels of HIV prevalence tend to have the smallest numbers of stable serodiscordant couples [77]: only a small minority (<15%) of infected individuals report being in a stable partnership with someone known to be uninfected [77]. There are few opportunities to identify serodiscordant couples in current health care systems in most settings in Africa, though household testing interventions may increase opportunities to reach couples [78]. However, many would question the general acceptability of an intervention that favours those in stable discordant partnerships over those in concordant partnerships. Operationally, defining a consistent criterion for a discordant couple is challenging. In Kenya, for example, there may be 150,000 individuals that would be newly eligible to start treatment today under this policy [77], but many more might claim to be in stable discordant relationships, or limitations in disclosure in couples could mean that many fewer would actually start treatment earlier.

### Female Sex Workers

Almost one-fifth of the HIV epidemics in sub-Saharan Africa are classified as concentrated (defined as HIV epidemics with HIV prevalence < 1% in the adult population), and many more are not highly disseminated (43% of epidemics in this region have an adult HIV prevalence below 3%) [79]. In these settings, FSWs and their clients are key populations for the transmission of HIV [80]–[86]. Previous modelling [81],[82],[87],[88] and epidemiological analyses [89],[90] suggest that prioritising interventions for FSWs and their clients in these settings can substantially reduce HIV transmission amongst FSWs, and amongst the population as a whole. It therefore seems natural to consider whether ART eligibility irrespective of CD4 cell count should be prioritised to FSWs.

A literature review was conducted in PubMed with the search terms “(“sex workers” or FSW or FSWs or CSW [commercial sex worker] or CSWs or sexwork*) and HIV and (antiretroviral or “anti-retroviral” or ART or HAART)”. This produced 67 papers, of which nine considered ART treatment amongst FSWs, barriers to accessing care, and risk behaviours following ART initiation [91]–[97]. The majority were from sub-Saharan Africa (six of nine), with three others from Chennai, India, and Vancouver, Canada. These papers informed the following discussion of ART prioritisation for FSWs.

In settings with existing and effective non-ART interventions to prevent HIV transmission amongst FSWs and their clients, the main questions are to evaluate the potential added prevention benefits of prioritising ART to FSWs, and the likelihood of risk substitution (i.e., potential increases in risk behaviours following ART expansion) [94],[98],[99]. In settings where behaviour-targeted interventions have not been fully implemented, the question is whether these should be scaled up before scaling up ART for prevention. If expanded access to ART is scaled up for FSWs following behaviour change interventions, this may increase the relative impact of ART treatment. For instance, because increases in condom use could reduce the incidence of new acute HIV infections, it is possible that such an intervention could temporarily lead to a smaller proportion of incident infections being due to early acute infection, and therefore a relatively greater prevention benefit of ART when provided to those with chronic infection.

Achieving a high preventive benefit from expanding ART to FSWs depends on initiating and retaining individuals in programmes. FSWs have generally received lower coverage of ART, because of factors such as reduced health-seeking behaviour and the stigmatised nature of sex work [100]. However, numerous targeted HIV prevention interventions worldwide show that FSWs can be engaged and recruited into intensive interventions with high coverage [101]–[103] and at reasonable cost [104]–[106]. Emerging data on ART provision amongst FSWs [92],[93],[95],[107] suggest that FSWs can be successfully initiated on ART in resource-poor settings; existing interventions could act as an easy and affordable entry point for increasing ART coverage among FSWs [93]. However, maintaining high ART adherence among FSWs remains challenging, leading to poorer outcomes with respect to CD4 count and suppression of viral load compared to non-FSWs [93],[107]. This is likely to translate into smaller reductions in infectivity, and greater morbidity or mortality [93],[107],[108], and indicates that there would be a particular need for retention efforts and adherence counselling for this prioritisation group [96], which could increase the costs of FSW-targeted ART programmes.

In addition to clinical and behavioural issues, the transient nature of sex work could affect the potential impact of ART on transmission, and the subsequent costs. As most FSWs sell sex for only a few years [109],[110], the early phase of acute HIV infection with high HIV viraemia may make a disproportionately large contribution to sex-work-driven HIV transmission [111]. Even with frequent retesting and immediate linkage to care, ART is likely to be initiated only after this phase, which would reduce the impact of ART on transmission, and highlights the continued importance of condom interventions. In addition, the cumulative costs of immediate ART eligibility for FSWs might grow sharply, as new FSWs become infected and eligible for ART, and HIV-infected former FSWs remain on ART after ceasing sex work. This could result in considerable investment and a suboptimal allocation of ART in some settings with rapid turnover of sex worker populations. However, it is possible that ex-FSWs may still be at greater risk of transmitting HIV than the general population and so could remain a good target population for ART as prevention.

Lastly, as has been shown for other HIV prevention interventions, the expected preventive benefit of targeting FSWs with ART is smaller in generalised epidemics than in concentrated epidemics, and is reduced in the late phase of an HIV epidemic compared to the early phase, especially in the short term. However, even in generalised HIV epidemics, modelling analyses suggest it would be cost-effective to target FSWs because of their disproportionate contribution to HIV transmission, although it may not be sufficient for achieving large and rapid reductions in HIV transmission in the general population. Conversely, not reaching high-risk groups such as FSWs may seriously attenuate the impact of any ART intervention [71],[112].

In summary, the decision to target FSWs with ART provision has to balance the likely heightened costs associated with increased adherence counselling and monitoring, and outreach to ensure retention, with benefits of decreased transmission that may be short-lived in contexts where sex work is transient. However, in settings where sex work is longer term the impact could be much greater. In addition, the ethical and social acceptability of giving prioritised ART access to FSWs needs to be carefully considered before any FSW-targeted programme is initiated—the benefits to the population as a whole would need to be clearly determined and communicated, and proactive monitoring of all ART provision channels would need to be in place to ensure that the care of other HIV-infected individuals is not compromised. Drug resistance should also be monitored, as FSWs on ART may facilitate the spread of resistance.

### Men Who Have Sex with Men and People Who Inject Drugs

The arguments for expanded access to other key populations, including MSM and PWID, are similar to those for expanded access to FSWs. If there is a population that contributes disproportionately to the number of infections in a population, and they can be identified and enrolled and retained in care, then it could be efficient to prioritise ART access to that group. However, the evidence from the HPTN 052 study that ART reduces infectiousness was specifically for heterosexual transmission: the extent to which ART decreases transmission occurring through homosexual sex or intravenous injection is not known [113].

Also, for the epidemics in Africa, there is little information about the population sizes of MSM and PWID, and their behaviours and contribution to the epidemic, which makes it hard to formulate firm recommendations about the benefits of prioritising access to these groups. Several studies in Africa have been able to recruit MSM [114],[115], and it has been estimated that, in total, transmission among MSM could account for 6% of new infections in Kenya and up to 21% in some concentrated epidemics [115], a range that is broadly supported by the Joint United Nations Programme on HIV/AIDS review of modes of transmission ([116]; K. K. Case, P. D. Ghys, E. Gouws, J. W. Eaton, P. Cuchi, et al., unpublished data]. Meanwhile, in a global review of injecting behaviour, there were no data (or estimated prevalence levels) for most African countries [117], and consequently the estimated number of HIV-infected PWID was very uncertain, ranging from 26,000 to 572,000 for sub-Saharan Africa. However, it has been hypothesized that in particular areas, such as Mombasa and Nairobi in Kenya, a high frequency of injecting among a growing population of PWID, coupled with overlapping sexual risk behaviours, has resulted in a substantial proportion of overall transmission possibly resulting from injection [118],[119]. There are also indications that PWID are less likely to access care and treatment services than others [120], and they have lower adherence [121] and retention to therapy [122],[123], so any ART programme prioritising ART to this group would presumably have to contend with these issues.

## Discussion

If it could be afforded, all HIV-infected individuals who wanted to initiate ART should be able to do so. However, resource constraints, at least in the short and medium term, necessitate some form of prioritising of HIV treatment through health policies. These policies should maximise epidemiological and clinical benefit while still being feasible, affordable, acceptable, and equitable [124]. To date, this prioritisation has been based principally on the CD4 cell count of HIV-infected individuals, as a marker of their immediate clinical need, but with the finding that ART reduces transmission risk, it is important to re-evaluate other ways in which ART could be allocated. In this article, we have examined several of the main options for prioritising ART access and have highlighted the key epidemiological and policy considerations that should guide decision-making and future research (summarised in Table 1).

**Table 1:** Likely profile of prevention and clinical impact, affordability, feasibility, and acceptability of alternative options for ART expansion beyond current guidelines.

Prioritisation Group	Impact on New HIV Infections	Impact on HIV-Related Morbidity and Mortality	Feasibility	Affordability	Acceptability
CD4 cell count (350–500 cells/µl)	− (Unlikely to be highly transmissible, relative to those at lower CD4 cell counts or other prioritisation groups)	? (Clinical trial evidence expected from START trial, reporting in 2015; unlikely to be as efficient as strategies targeting clinical need, e.g., high SPVL, TB coinfection)	+ (Screening utilises already standard CD4 screening; reaching high coverage would likely require efforts to improve routine HIV testing at the population level)	− (Would likely expand access to treatment to an additional 20% of the HIV-infected populations)	+ (May be perceived as the most equitable option for expanding access to ART, because of the history of determining treatment need and access based on CD4 cell count)
Viral load (SPVL≥50,000 copies/ml)	• (Strong evidence from many discordant studies that infectiousness increases with SPVL, but not dramatically)	+ (Strong evidence from many seroconverter cohorts that individuals with high SPVL progress rapidly to AIDS, and so may enhance linkage to care in rapid progressors)	? (Requires development of point-of-care viral load testing; many prototypes, but none validated yet)	? (Cost of point-of-care viral load testing is unknown)	? (May prove controversial if not backed by evidence for direct clinical benefit)
Active TB disease	− (Likely to have the same impact on HIV transmission as reaching a subset of HIV-infected individuals with CD4 cell counts between 350 and 500 cells/µl)	+ (Much greater impact on morbidity and mortality than treating many other groups)	+ (Can be integrated with existing TB services; adherence/retention to ART may be higher because of current illness and the prospect of a reduced risk of TB recurrence)	+ (Relatively small group, compared with individuals with CD4 350–500 cells/µl; large reduction in mortality suggests targeting TB patients may be more cost-effective than other groups)	+ (Given the clear clinical need, likely to be highly acceptable to both the target group and the general population)
Pregnant women	? (Potential reductions in maternal orphanhood and potential to prevent paediatric HIV transmission; estimates of the impact on heterosexual HIV transmission are yet to be produced)	+ (Impact mainly on morbidity and mortality of newborns with HIV-positive mothers)	+ (Targets are easy to identify via existing ANC; testing uptake is high in some areas and can be increased by provider-initiated service; contrary results are found on retention)	+ (Increment of newly identified target patients is not big; infrastructures and staff that already exist favours the affordability)	+ (May be better accepted by patients if initiated by ANC provider)
Serodiscordant couples	− (Likely fewer infections averted per person-year of ART than allocation to those with multiple partners)	? (Marginal therapeutic benefit of ART initiation at CD4 >350 cells/µl not certain)	+ (In some settings couples hard to find; trial data indicate discordant couples are a highly motivated population with good adherence to pill-taking regimes and retention in care)	? (Minority of infected individuals in stable discordant couples, but uptake unknown)	? (Unclear if it is socially acceptable for those with stable partners to receive preferential access)
Sex workers	+ (Elevated HIV transmission risk in many settings likely to result in large number of HIV infections averted per year on ART)	? (May be more modest than other groups because limited data suggest that they have lower adherence and worse outcomes in terms of morbidity and mortality)	+ (Previous FSW-targeted interventions have demonstrated feasibility; limited studies suggest FSWs are willing to initiate ART; however, likely to have worse adherence and retention)	+ (FSWs make up a small proportion of the female population, but if sex work is of short duration, then there may be a much larger group of ex-FSWs that will continue on ART)	? (May not be acceptable to the wider community; programmes would need to show and emphasise population benefit)

?, insufficient evidence to warrant definitive decision; +, available evidence suggests this is a beneficial option, compared to the other expansion options; −, available evidence suggests this is an unfavourable option, compared to the other expansion options; •, available evidence supports neither that this is a beneficial option nor that it is an unfavourable option.

There are some forms of prioritisation that are already supported by existing guidelines or programmes. In particular, ART for all individuals with active TB disease has substantial epidemiological and clinical benefits, and already forms part of WHO international guidelines. Treatment for pregnant women irrespective of CD4 cell count, for which the epidemiological impact is not yet clear, could have advantages in terms of simplified care for pregnant women and benefits for their children and partners, and is being implemented in some settings.

Important questions remain regarding all of the options, and there is a clear need for further data collection. Some knowledge gaps could be filled shortly, as results are reported from at least 50 projects planned or ongoing to evaluate the impact of ART and other interventions on HIV- and/or TB-related morbidity and mortality, HIV incidence and transmission, and risk behaviour [125]. Almost half of these projects are in sub-Saharan Africa, and include studies that will test the individual- and community-level preventive effect of ART provided to patients with CD4 cell counts between 350 and 500 cells/µl, those with the highest viral loads, HIV-infected pregnant women, and HIV-infected partners in serodiscordant couples [125],[126]. In addition, the secondary objectives of many of these projects are the evaluation of the feasibility, cost, health care impact, treatment adherence and retention, and social acceptability of the interventions. With a large variation in geographical areas, target populations, and outcome variables, the combined body of evidence generated by these studies may begin to address the question of whether and how different sociodemographic, economic, and epidemiological contexts influence the impact of ART interventions.

In the short term, the costs of expanding access to ART are likely to be driven by the size of the groups to whom access is extended and the costs associated with identifying and reaching members of these groups. Long-term affordability is likely to depend on the size of the group as well as reductions in incidence resulting from the expanded ART programme, the success of other HIV prevention interventions, and economic nonlinearities such as economies of scale. Although relative group sizes will vary from setting to setting, ART expansion to HIV-infected people with CD4 counts of 350–500 cells/µl or above 500 cells/µl would likely require the largest programme increase. In contrast, initial increases required to prioritise ART in FSWs and pregnant women would most likely be much smaller than for the other prioritisation options, though cumulative costs would grow as women started ART during pregnancy or sex work, then continued on lifelong ART.

Evidence from ART programmes in southern Africa indicates that high retention in care becomes increasingly challenging as treatment programmes expand [127]. Affordability and feasibility are negatively affected not only by larger group size, but also by the more intensive efforts required to identify eligible people and maintain high adherence and retention in care. Globally, patients' health literacy regarding ART adherence remains an important challenge [128]. On the other hand, there is a rapidly growing body of strategies and tools to improve retention in care and ART adherence, including interventions to improve the mental health (especially treatment of depression) of HIV-positive individuals, and reminder devices and interactive communication technologies [129].

This review has aimed to highlight some of the key issues and identify the needs for future studies, and has not provided a direct quantitative comparison of the impact of alternative prioritisation strategies in specific settings, which will be a critically important body of modelling work in the future. To further facilitate a constructive debate that is meaningful to national decision-makers and donor organisations, context-specific mathematical models should be developed that enable head-to-head comparison of multiple ART expansion options in an internally consistent manner, that is, with all simulations based on the same data and assumptions. However, the considerations raised here already indicate that the impact and feasibility of these alternative forms of ART allocation are expected to vary substantially between settings, and there is no single formulation that will be optimal in all settings. Furthermore, the best strategy will depend on the relative values assigned to therapeutic benefits, preventative benefits, and wider societal benefits, such as reducing the number of orphans and increasing labour force availability. Combined metrics of impact such as quality-adjusted life years saved or disability-adjusted life years averted [130] can be used to understand how preventative and therapeutic benefits are related.

The overall effectiveness of treatment in reducing infectiousness, as well as the risk of drug resistance [130], is expected to be crucially dependent on the viral suppression achieved, which is in turn affected by patterns of adherence. Throughout this analysis, we have assumed ART has a suppressive effect on HIV transmission for all patients receiving the treatment. Whilst this is biologically plausible, we recognise that it is possible that different groups could behave differently from the HIV-infected individuals in the HPTN 052 trial and therefore achieve lower levels of viral suppression and a smaller reduction in infectiousness. However, there is little information available on levels of viral suppression for ART, nor on adherence to the treatment regimen.

Finally, we recognise that the issues of expanding access to ART do not exist in a vacuum. Decisions concerning the implementation of ART should be scaled—scaling up will have to take place in the context of the entire portfolio of the HIV response programme in a particular country, which will include multiple forms of prevention intervention. Indeed, WHO guidance on the use of antiretrovirals for prevention is expected to include both pre-exposure prophylaxis and ART, and we would anticipate that further strategic advice from normative agencies will increasingly embrace the full range of possibilities for maximising the health impact of ART in combination with other interventions [131].

**Key Points**

- Discussions about expanded access to ART for HIV prevention have been focused on one particular strategy—providing ART to all HIV-infected individuals. Here we aim to broaden the discussion by considering the implications of prioritising access to ART according to clinical and behavioural factors.
- Any recommendation to prioritise particular groups should consider not only the impact of ART in that group, including its therapeutic and prevention effects, but also its feasibility, affordability, and acceptability.
- Some forms of prioritisation—ART for individuals with active TB and for pregnant women irrespective of CD4 cell count—are already promoted by existing guidelines or programmes.
- For other prioritisation options, there are currently insufficient data to make first recommendations, although findings of future studies and further modelling analyses should contribute to forming policy.

## Abbreviations

ANC: antenatal care
ART: antiretroviral therapy
FSW: female sex worker
MSM: men who have sex with men
PEPFAR: US President's Emergency Plan for AIDS Relief
PMTCT: prevention of mother-to-child transmission
PWID: people who inject drugs
SPVL: set-point viral load
TB: tuberculosis
WHO: World Health Organization
